# Leaf photosynthetic characteristics of waxy maize in response to different degrees of heat stress during grain filling

**DOI:** 10.1186/s12870-023-04482-7

**Published:** 2023-10-06

**Authors:** Lingling Qu, Xiaotian Gu, Jing Li, Jian Guo, Dalei Lu

**Affiliations:** 1 Jiangsu Key Laboratory of Crop Genetics and Physiology, Jiangsu Key Laboratory of Crop Cultivation and Physiology, Jiangsu Co‒Innovation Center for Modern Production Technology of Grain Crops, Agricultural College of Yangzhou University, Yangzhou, 225009 China; 2 Yancheng Institute Of Technology, Yancheng, 224000 China; 3 Joint International Research Laboratory of Agriculture and Agri‒Product Safety, the Ministry of Education of China, Yangzhou, 225009 China

**Keywords:** Heat stress, Waxy maize, Photosynthetic, Antioxidant enzymes, Yield

## Abstract

**Background:**

In the context of climate change, maize is facing unprecedented heat stress (HS) threats during grain filling. Understanding how HS affects yield is the key to reducing the impact of climate change on maize production. Suyunuo5 (SYN5) and Yunuo7 (YN7) were used as materials, and four temperature gradients of 28℃ (day)/20℃ (night; T0, control), 32 °C/24°C (T1, mild HS), 36 °C/28°C (T2, moderate HS), and 40 °C/32°C (T3, severe HS) were set up during grain filling to explore the physiological mechanism of different degrees HS affecting photosynthetic characteristics of leaves in this study.

**Results:**

Results showed that HS accelerated the degradation of chlorophyll, disturbed the metabolism of reactive oxygen species, reduced the activity of antioxidant enzymes, and caused leaf damage. Heat stress induced the down-regulation of photosynthesis-related genes, which results in the decrease of enzymatic activities involved in photosynthesis, thereby inhibiting photosynthesis and reducing yield. Integrated analysis showed that the degree of the negative influence of three HS types during grain filling on leaves and yield was T3 > T2 > T1. The increase in HS disturbed leaf physiological activities and grain filling. Meanwhile, this study observed that the YN7 was more heat tolerance than SYN5 and thus it was recommended to use YN7 in waxy maize planting areas with frequent high temperatures.

**Conclusions:**

Heat stress during grain filling caused premature senescence of the leaves by inhibiting the ability of leaves to photosynthesize and accelerating the oxidative damage of cells, thereby affecting the waxy maize yield. Our study helped to simulate the productivity of waxy maize under high temperatures and provided assistance for a stable yield of waxy maize under future climate warming.

**Supplementary Information:**

The online version contains supplementary material available at 10.1186/s12870-023-04482-7.

## Background

Global average temperatures continuously increase over time due to the increasing climate change, despite efforts by certain nations to attain net-zero carbon emissions, such as China’s ‘double carbon’ target [1]. Global warming will likely reach 1.5 °C between 2030 and 2052 based on multi-model simulations, which further increases the frequency and intensity of climate extremes, thereby causing serious damage to agricultural production [2, 3]. Maize (*Zea mays* L.), an important cereal crop worldwide, is severely threatened by extreme heat stress (HS) [4, 5]. Extreme HS substantially diminishes the photosynthetic rate of the leaves, lengthens the anthesis-silking interval, raises grain abortion, decreases filling time, and finally causes irreversible maize yield losses [6].

Heat stress during grain filling affects the source and sink of maize, resulting in low and unstable grain yield and quality [7, 8]. Source capacity is directly affected by the decline in carbohydrate synthesis caused by a decrease in photosynthesis and an increase in respiration rate [9]. Photosynthesis is a vital physiological process in plants, which largely determines the growth and productivity of plants, and this process is greatly susceptible to temperature changes [10]. When exposed to HS and intense light, a photosynthetic apparatus is extremely susceptible to harm [11]. The efficiency of photosynthesis decreases during leaf senescence, which is a primary physiological change [12]. Chlorophyll production is inhibited by HS, and reactive oxygen species (ROS) build up in the leaves, increasing the breakdown of thylakoid components and accelerating leaf senescence [13]. In general, plants activate defensive mechanisms to preserve metabolic balance whilst under stress, but HS decreases the activity of antioxidant enzymes, preventing them from effectively removing ROS and preserving cell homeostasis [14]. Heat stress also disrupts the hormone balance in the leaves, which has an additional negative effect on their structure, accelerates senescence, and reduces source activity [15]. Numerous genes also control the performance of leaves during photosynthesis. During the regulation of C4 cycle under HS, genes expression of related enzymes are inhibited, and enzymatic activity diminishes, thereby reducing the supply of CO_2_ involved in the Calvin cycle, leading to a decrease in leaf photosynthetic capacity [16]. For example, the ribulose bisphosphate carboxylase (RuBPCase), a key enzyme in photosynthesis, undergoes a cascade effect of HS during flowering, which down-regulates the expression of related genes, reducing its activity and inhibiting the photosynthetic rate [17]. Therefore, HS leads to yield loss by limiting carbon assimilation and electron transfer, cell oxidative damage, and photoinhibition of photosystem II [18].

Waxy maize (*Zea mays* L. *var. ceratina Kulesh*), which is abundantly grown in China, is a favourite food in South Asia [19, 20]. In addition, waxy maize starch has a high viscosity, and it is easy to digest because of its high content of amylopectin, which makes it popular in the food and non-food industries [21]. Thus, waxy maize has great application potential as a global economic potential crop. Nevertheless, HS encountered during grain filling in the production of waxy maize can accelerate senescence in the leaves, which further affects grain yield, quality and economic advantages. Hence, elucidating the physiological mechanism of leaves in response to HS is important to improve the yield of waxy maize. In this study, changes in photosynthetic performance, antioxidant enzymatic activities, endogenous hormone contents, and grain yield of two waxy maize varieties under different degrees of HS during grain filling were compared. This study revealed the physiological mechanism of leaf response to HS during grain filling and will provide an important mitigation plan for the high-yield cultivation of waxy maize in future climate change.

## Results

### Chlorophyll content

**Fig. 1:**
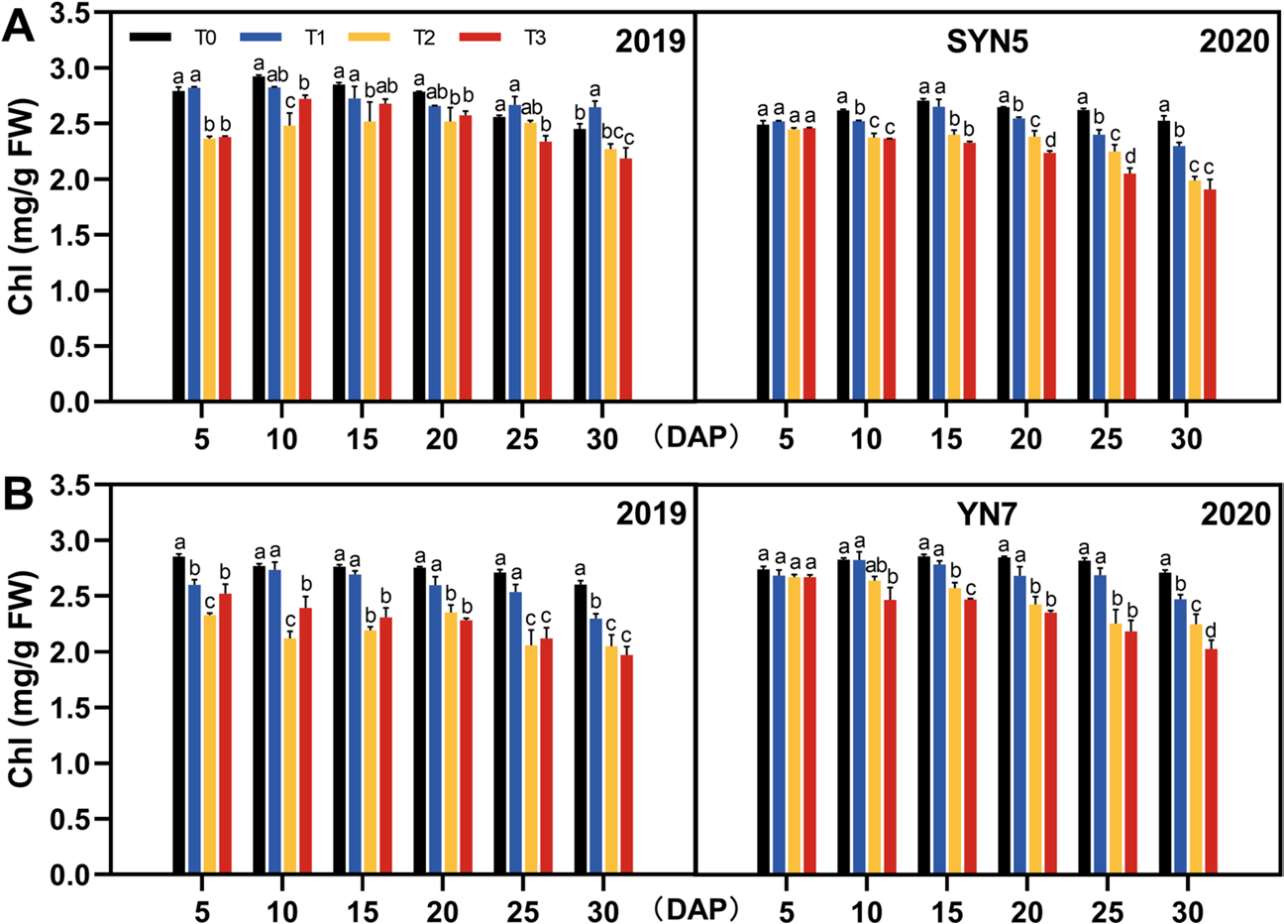
Effect of HS during grain-filling stage on chlorophyll content in waxy maize leaves. **A**, SYN5; **B**, YN7. Chl, chlorophyll; T0, control; T1, mild HS; T2, moderate HS; T3, severe HS. The error bars indicate standard errors (*n* = 3 replicates), and letters indicate significant difference (*P* < 0.05) at the same sampling date

Three degrees of HS treatments had significant effects on chlorophyll content in the leaves. Compared with the control (T0), the chlorophyll content reduced in the leaves as the temperatures increase from T1 to T3. During grain filling, the chlorophyll content of SYN5 leaves in both years decreased on average by 2.1%, 10.7% and 11.8% under T1, T2, and T3 treatments when compared to T0, respectively (Fig. 1A). Similarly, YN7 decreased by 5.1%, 16.3% and 16.6%, respectively (Fig. 1B). The chlorophyll content in the leaves of both waxy maize varieties showed the greatest decrease under the influence of severe HS (T3).

### Photosynthetic parameters

**Fig. 2:**
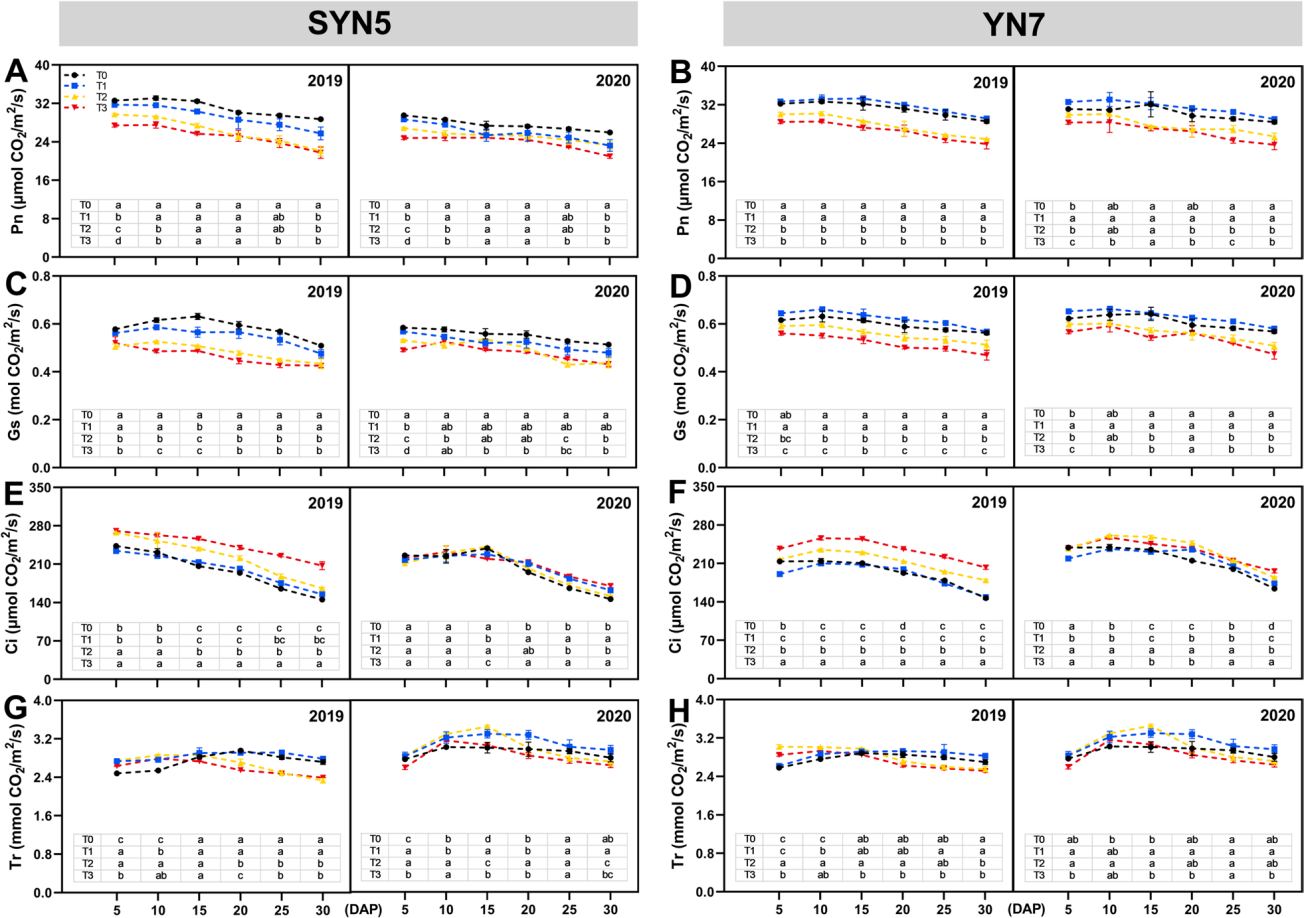
Effect of HS during grain-filling stage on photosynthetic characteristics of waxy maize leaves. **A**, **B** Pn, photosynthetic rate; **C**, **D** Gs, stomatal conductance; **E**, **F** Tr, transpiration rate; **G**, **H** Ci, intercellular CO_2_ concentration. T0, control; T1, mild HS; T2, moderate HS; T3, severe HS. The error bars indicate standard errors (*n* = 3 replicates), and letters indicate significant difference (*P* < 0.05) at the same sampling date

Heat stress has affected the photosynthetic characteristics of the leaves of two waxy maize varieties. The T1, T2 and T3 treatments resulted in an average decrease of 6.0%, 12.1% and 16.3% in net photosynthetic rate (Pn) of SYN5, respectively (Fig. 2A). Likewise, compared to the T0 treatment, T2 and T3 treatments of YN7 decreased Pn by 9.5% and 13.6%, respectively, while T1 treatment increased Pn by 3.2% (Fig. 2B). The Pn of YN7 leaves illustrated lesser reductions at HS treatments. Under various HS treatments, the Gs of the two maize varieties showed a similar trend to Pn (Fig. 2C and D). Moreover, the T2 and T3 treatments improved Ci of SYN5 and YN7 by 7.1%, 15.3% and 9.7%, 15.5%, respectively (Fig. 2E and F). Compared with the T0 treatment, as the grouting process progressed, the Tr under HS first increased and then decreased. Under T2 and T3 treatments of SYN5, Tr increased by 13.5% and 8.1% individually during early grain filling but decreased by 4.6% and 6.1% in the later stage (Fig. 2G). Equally, under T2 and T3 treatments, the Tr of YN7 was enhanced by 9.3% and 2.6% at early filling stage, respectively, and diminished by 4.1% and 6.7% at the later filling stage (Fig. 2H).

### Photosynthesis-related enzymatic activity

**Fig. 3:**
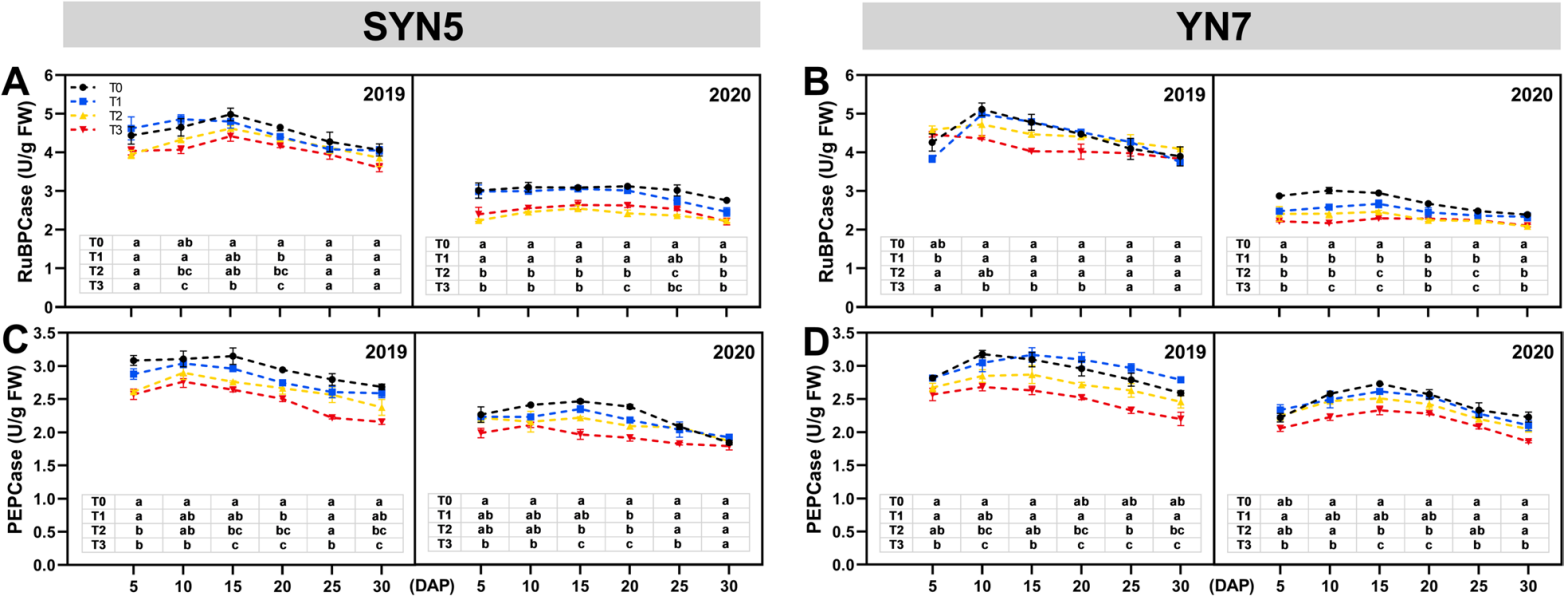
Effect of HS during grain-filling stage on RuBPCase and PEPCase activities of waxy maize leaves. **A**, **B** RuBPCase, ribulose bisphosphate carboxylase; **C**, **D** PEPCase, phosphoenolpyruvate carboxylase. T0, control; T1, mild HS; T2, moderate HS; T3, severe HS. The error bars indicate standard errors (*n* = 3 replicates), and letters indicate significant difference (*P* < 0.05) at the same sampling date

The activities of RuBPCase and phosphoenolpyruvate carboxylase (PEPCase) in SYN5 and YN7 decreased during grain filling when the plants underwent post-silking HS (Fig. 3). Compared with T0 treatment, T2 treatment reduced RuBPCase activities of SYN5 and YN7 on average by 13.9% and 7.5%, whilst T3 treatment decreased RuBPCase activities of SYN5 and YN7 by 13.9% and 12.4%, respectively (Fig. 3A and B). Similarly, PEPCase activities of SYN5 and YN7 were repressed by 8.1% and 6.1% in T2 and 14.9% and 13.4% in T3, respectively (Fig. 3C and D). The decline ratio of photosynthetic enzymatic activity of YN7 leaves under HS was lower than that of SYN5.

### Expression of genes related to photosynthesis

**Fig. 4:**
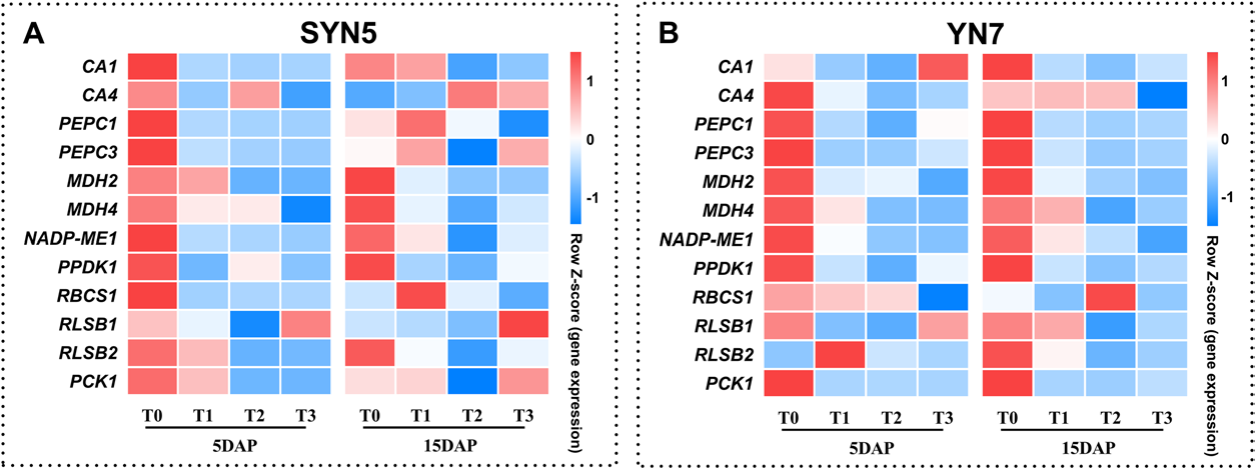
Effect of HS during grain-filling stage on the expression of photosynthesis-related genes. T0, control; T1, mild HS; T2, moderate HS; T3, severe HS. The error bars indicate standard errors (*n* = 3 replicates), and letters indicate significant difference (*P* < 0.05) at the same sampling date

In verifying the impact of HS on the transcript level of leaf photosynthesis, our study selected 12 genes encoding carbonic anhydrases (*CA1* and *CA4*), phosphoenolpyruvate carboxylase (*PEPC1* and *PEPC3*), malate dehydrogenase (*MDH2* and *MDH4*), NADP-dependent malic enzyme (*NADPH-ME1*), RuBPCase (*RBCS1*, *RLSB1*, and *RLSB2*), pyruvate orthophosphate dikinase (*PPDK1*), and phosphoenolpyruvate carboxykinase (*PCK1*) for qRT-PCR analysis. The *CA1*, *PEPC1*, *PEPC3*, *MDH4*, *NADPH-ME1*, *PPDK1*, and *RBCS1* genes were down-regulated in SYN5 at 5 days after pollination (DAP) under three HS treatments (Fig. 4A). However, some genes (*CA4*, *PEPC1*, *PEPC3*, *RBCS1*, and *PCK1*) were up-regulated in different degrees on the 15th day (15 DAP) after HS of SYN5. The *CA4*, *PEPC1*, *PEPC3*, *MDH2*, *MDH4*, *NADPH-ME1*, and *PPDK1* genes were down-regulated in YN7 at 5 DAP under three HS treatments (Fig. 4B). Except for *CA4*, *MDH4*, *RBCS1*, and *RLSB1*, all other genes were down-regulated in YN7 at 15DAP.

### Chlorophyll fluorescence parameters

Under T1 treatment during grain filling, the chlorophyll fluorescence characteristics of the two varieties did not change substantially. Compared with T0 treatment, T2 treatment reduced the apparent electron transfer rate (ETR) of SYN5 and YN7 by 4.4% and 5.2%, respectively, whereas T3 treatment decreased the ETR of SYN5 and YN7 by 14.4% and 18.3%, respectively (Fig. S2 A and B). At the same time, photochemical quenching (qP) of the two varieties primarily tended to rise in the early stage and fall significantly in the later stage under HS treatment (Fig. S2, C and D). During the HS treatment period, non-photochemical quenching (NPQ) showed an overall tendency to increase with the increase of temperature, reaching a maximum under T3 treatment. T3 treatment improved the NPQ of SYN5 and YN7 by 8.2% and 16.3%, respectively, in comparison to T0 treatment (Fig. S2, E and F). Furthermore, under T2 and T3 treatments, the photosystem II (PSII) primary maximum light energy use efficiency (Fv/Fm) of SYN5 and YN7 diminished by 9.2%, 11.6% and 9.6%, 12.6%, respectively. The results of the two-year experiment showed that the overall Fv/Fm of PSII under T2 and T3 treatments was significantly lower than that of T0 treatment, and no recovery was observed at the end of treatment (Fig. S2, G and H).

### Soluble protein and sugar content

During grain filling in 2 years, the content of soluble protein and sugar in leaves was reduced by HS, and significant changes were observed under T1 and T2 treatments (Fig. S3). The T2 treatment declined the soluble protein content of SYN5 and YN7 by 8.8% and 7.1%, whereas T3 treatment decreased the soluble protein content of SYN5 and YN7 by 17.8% and 11.9%, respectively, in comparison to T0 treatment (Fig. S3 A and B). Additionally, compared with T0 treatment, T2 treatment reduced the soluble sugar content of SYN5 and YN7 by 6.2% and 17.2%, whereas T3 treatment decreased the soluble sugar content of SYN5 and YN7 by 12.0% and 14.3%, respectively (Fig. S3 C and D).

### Malondialdehyde (MDA) and ROS content

**Fig. 5:**
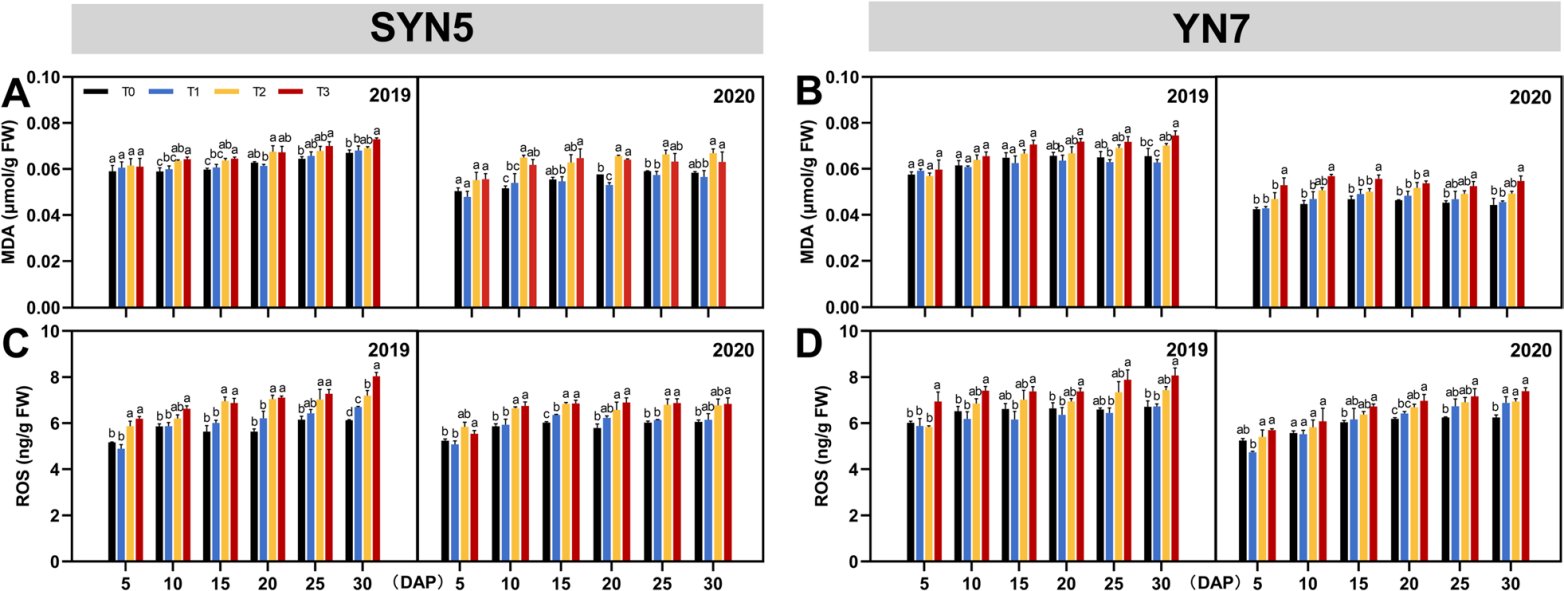
Effect of HS during grain-filling stage on MDA and ROS contents of waxy maize leaves. **A**, **B** MDA, malondialdehyde; **C**, **D** ROS, reactive oxygen species; T0, control; T1, mild HS; T2, moderate HS; T3, severe HS. The error bars indicate standard errors (*n* = 3 replicates), and letters indicate significant difference (*P* < 0.05) at the same sampling date

With post-silking HS treatment during grain filling, MDA and ROS contents in the leaves of both varieties increased, except for T1 treatment (Fig. 5). The MDA content was increased by 10.3%, 9.8% and 6.8%, 14.8% in SYN5 and YN7 under the influences of T2 and T3 treatments as compared to T0. Similarly, the ROS content of both varieties was increased by 7.7%, 16.6% and 7.2%, 9.9% under T2 and T3 treatments, respectively. The ROS accumulation in SYN5 leaves was higher than that in YN7 under HS.

### Antioxidant enzymatic activity

**Fig. 6:**
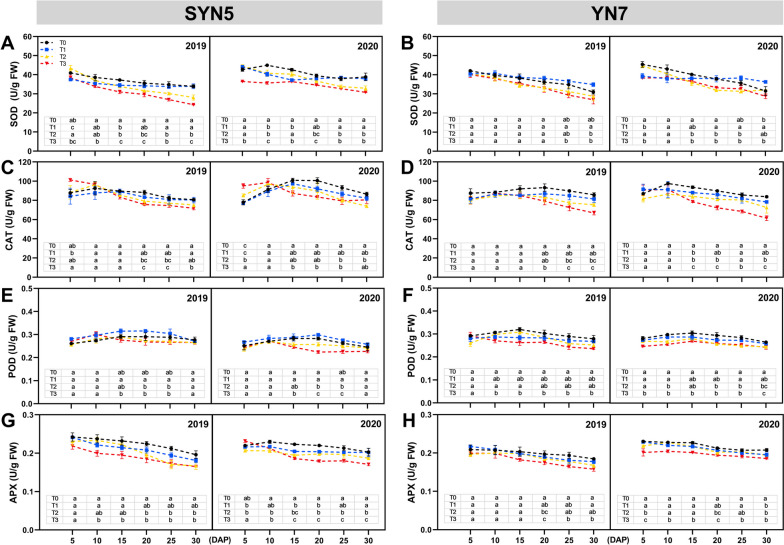
Effect of HS during grain-filling stage on leaf antioxidant enzymes of waxy maize. **A**, **B** SOD, superoxide dismutase; **C**, **D** POD, peroxidase; **E**, **F** CAT, catalase; **G**, **H** APX, ascorbate peroxidase. T0, control; T1, mild HS; T2, moderate HS; T3, severe HS. The error bars indicate standard errors (*n* = 3 replicates), and letters indicate significant difference (*P* < 0.05) at the same sampling date

The activities of antioxidant enzymes in the leaves of both waxy maize varieties were down-regulated under T2 and T3 treatments during grain filling (Fig. 6). Under the T2 and T3 treatments, the superoxide dismutase (SOD) activity of SYN5 and YN7 was decreased by 7.7%, 16.6% and 7.2%, 9.9% compared with T0 treatment, respectively (Fig. 6A and B). The catalase (CAT) and peroxidase (POD) activity showed a similar reduction pattern to SOD, and the decrease became greater with the increase in temperature (Fig. 6C, D, E, and F). In addition, the ascorbate peroxidase (APX) activity of SYN5 and YN7 was repressed by 5.5%, 9.4%, 13.5% and 2.7%, 4.9%, 10.1% under the influences of T1, T2 and T3 treatments, respectively, compared with T0 treatment (Fig. 6G and H). Both maize varieties showed the greatest decrease in antioxidant enzymatic activity under T3 treatment. In SYN5 leaves under stress, the activity of antioxidant enzymes decreased more than in YN7 leaves. Therefore, under the influences of most heat treatments, the antioxidant enzyme activities of SYN5 decrease more than that in YN7.

### Auxin (IAA) and abscisic acid (ABA) content

**Fig. 7:**
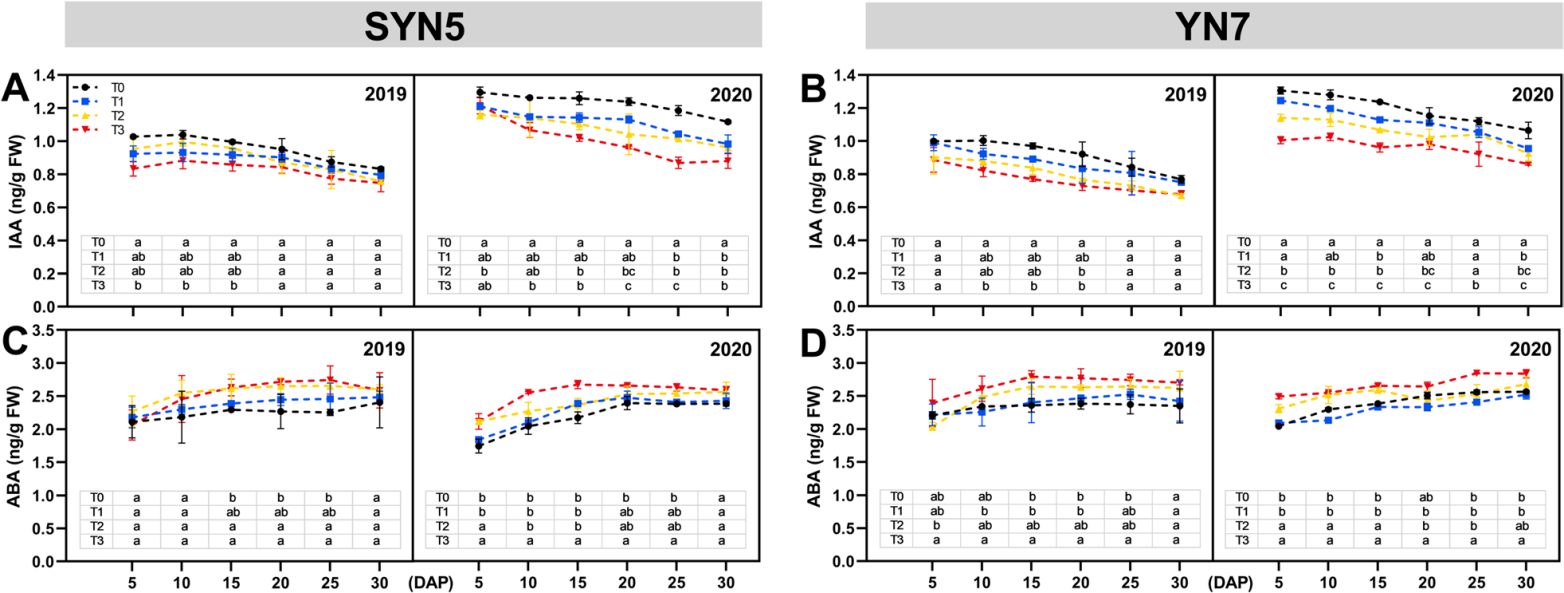
Effect of HS during grain-filling stage on IAA and ABA contents of waxy maize leaves. **A**, **B** IAA, auxin; **C**, **D** ABA, abscisic acid. T0, control; T1, mild HS; T2, moderate HS; T3, severe HS. The error bars indicate standard errors (*n* = 3 replicates), and letters indicate significant difference (*P* < 0.05) at the same sampling date

The three HS treatments reduced the IAA content and increased the ABA content of both waxy maize varieties during grain filling when compared with the control (Fig. 7). Compared with T0 treatment, the IAA content of SYN5 and YN7 decreased during T1, T2 and T3 treatments by 8.3%, 9.5%, 16.0% and 6.1%, 12.3%, 18.0%, respectively (Fig. 7A and B). The content of ABA in SYN5 under T1, T2 and T3 treatments was increased by 4.8%, 12.1% and 14.5% respectively. Similarly, the ABA of YN7 increased by 6.3% and 13.1% compared to the control under T2 and T3 treatments, respectively. On the contrary, under T1 treatment, the ABA of YN7 decreased by 0.8% (Fig. 7C and D). Comprehensive investigation reveals that the hormone content changes more, and SYN5 is more affected when the degree of HS aggravates.

### Yield

**Fig. 8:**
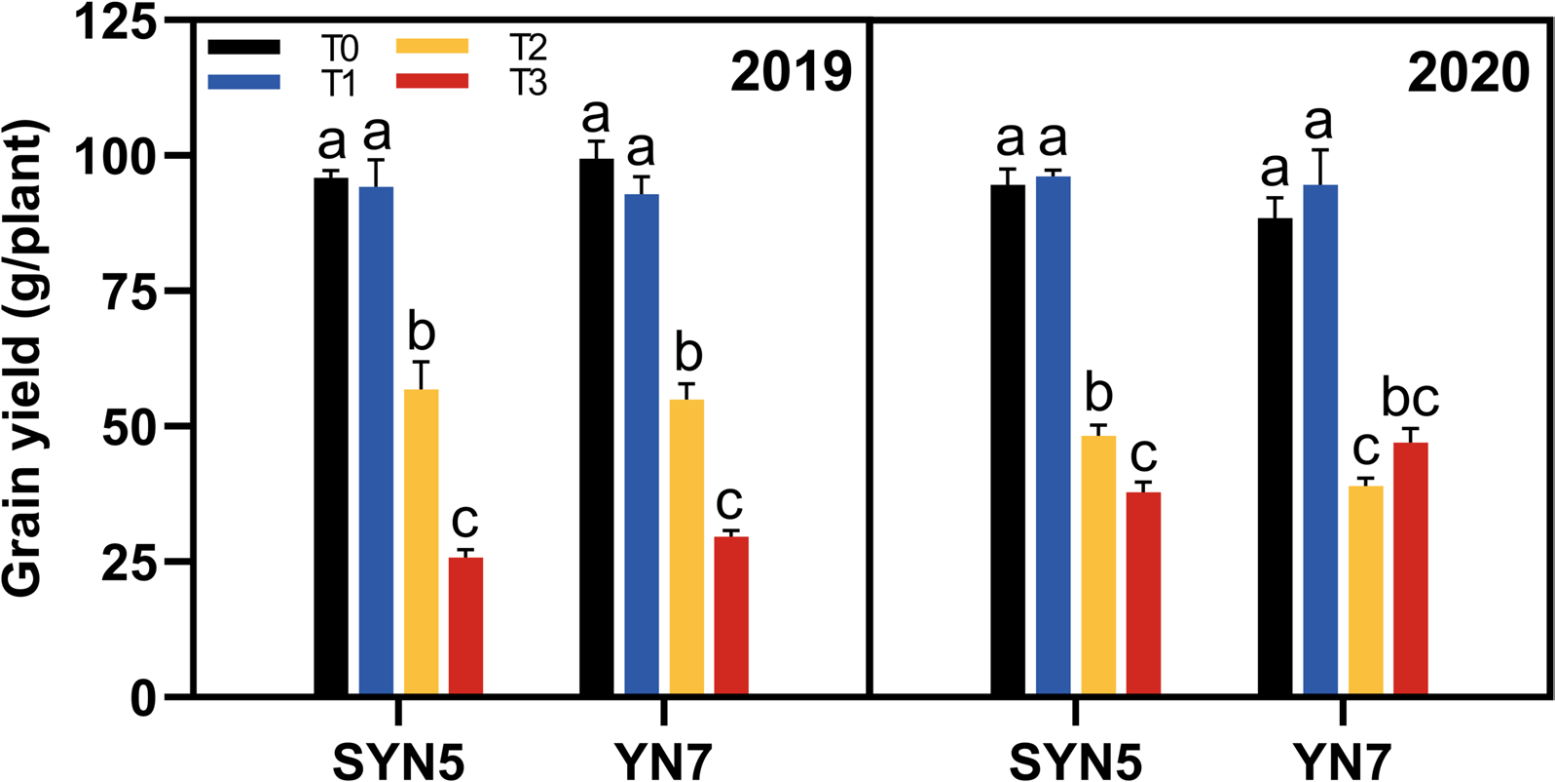
Effect of HS during grain-filling stage on grain yield of waxy maize. T0, control; T1, mild HS; T2, moderate HS; T3, severe HS. The error bars indicate standard errors (*n* = 3 replicates), and letters indicate significant difference (*P* < 0.05) at the same variety

The yield changes of waxy maize were different under different HS conditions, and the yield reduction of SYN5 was greater than that of the YN7 (Fig. 8). Compared with T0 treatment, the average yield of SYN5 and YN7 in both years decreased by 44.9% and 50.3% under T2 treatment, as well as 66.6% and 58.5% under T3 treatment, respectively, but no significant difference was observed under T1 treatment (*P* < 0.05). Based on the results, except for the largest reduction in the production of YN7 under T2 treatment in 2020, all others experienced the largest reduction under T3 treatment.

## Discussion

The growth and development of plants are affected by HS due to the significant reduction in leaf photosynthesis. The first step in photosynthesis is the light’s absorption, particularly by chlorophyll molecules [22]. As a result, the amount of chlorophyll in a leaf greatly influences its ability to absorb light. Heat stress decreases the chlorophyll content, reduces the interception of light quanta and decreases the net photosynthetic rate, thereby resulting in lower grain yield [23]. The current study demonstrated that HS accelerated chlorophyll degradation, causing leaves to chlorosis, thereby limiting their ability to capture and utilise light energy as well as impairing photosynthesis (Figs. 1, 2 and 8). This study found that leaves were adversely harmed by severe HS. The PS II found in thylakoid membranes of the chloroplast is extremely sensitive to HS. In the presence of thylakoid membrane damage, electron transfer and adenosine triphosphate phosphate synthesis are diminished, along with changes in photochemical reactions [24]. Our study showed that moderate and severe HS significantly reduced chlorophyll fluorescence parameters (Fv/Fm, ETR and qP) whilst substantially increasing NPQ, which agrees with earlier findings results in wheat, potato, tomato and other crops [22, 25].

In the present study, Pn, Gs and Tr decreased because of HS whilst Ci increased, indicating that the effect of HS on the photosynthetic rate was limited by stomatal and non-stomatal factors (Fig. 2). The comparison among varieties showed that the photosynthetic rate of YN7 showed smaller limitations. Ribulose bisphosphate carboxylase plays a crucial role in photosynthetic carbon assimilation in crops. Heat stress reduces the activity of RuBPCase by down-regulating the expression level of coding gene, thereby limiting photosynthesis [17]. The C4 carbon absorption cycle is disrupted by HS, and transcript abundance and activity of related enzymes (PEPCase, NADP-ME and MDH) are markedly decreased [16]. Similar to earlier research, HS down-regulated the expression level of genes encoding ribulose bisphosphate carboxylase (*RBCS1*, *RLSB1* and *RLSB2*), phosphoenolpyruvate carboxylase (*PEPCase1* and *PEPCase3*), NADP-malic enzyme (*NADP-ME1*) and NADP-malate dehydrogenase (*MDH2* and *MDH4*). Pyruvate orthophosphate dikinase that converts CO_2_ in the air into HCO_3_^−^, which can be fixed by PEP, and carbonic anhydrase that promotes the regeneration of PEP also play a crucial role in C4 carbon absorption cycle [26]. The *CA1*, *CA2* and *PPDK1* genes encoding carbonic anhydrase and pyruvate orthophosphate dikinase were down-regulated at HS based on our findings (Fig. 4). Heat stress affects the C4 pathway and down-regulates the expression level of associated genes, therefore limiting the supply of CO_2_ to Rubisco, reducing the production of photosynthates and ultimately resulting in a decline in production.

Senescence is the last phase of organ development in plants, which typically leads to programmed cell death [27]. Chlorophyll degradation and photo-oxidative stress are essential hallmarks of senescence in the leaves, which lead to the decrease of photosynthetic capacity and the production of ROS [28]. Under HS treatment, as ROS metabolism becomes imbalanced and damage occurs to the cell membrane, lipid peroxidation in the membranes and permeability increase, leading to the accumulation of MDA in leaf cells [29]. This study found that HS increased MDA and ROS contents in the leaves during grain filling, particularly at severe HS. In addition, this study found that severe HS had a stronger inhibitory effect on SOD, POD, CAT and APX activities, further exacerbating the oxidative damage and senescence of leaves, with a more severe impact in SYN5. Correlation analysis also proved that the oxidative damage of the leaves under HS treatment led to a decline in photosynthetic performance (Fig. S4).

Changes in the levels of osmotic substances during HS, such as carbohydrates, proteins and amino acids, are indicators of plant homeostasis regulation, and their accumulation is beneficial to the stability of the internal environment [30]. Decreasing the osmotic potential of cell protoplasm, such substances induce cells to absorb water from the outside, thereby maintaining a certain water content and expansion pressure of cells and mitigating the damage of cell dehydration to the plant body under stresses [31, 32]. In this study, a significant decrease in soluble sugar and soluble protein contents in the leaves of both waxy maize varieties was observed under moderate and severe HS treatments, which indicates that leaves were severely damaged, and the internal environment was extremely unstable (Fig. S3). Correlation analysis showed that the soluble protein content of leaves was significantly and positively correlated with Pn and POD as well as negatively correlated with MDA and ROS under HS (Fig. S4). The above-mentioned results indicate that HS during grain formation disrupts cell membrane integrity and metabolic processes, leading to leaf senescence. Moreover, soluble protein, ROS, MDA content and APX activity of YN7 leaves under HS were less affected by fluctuations than SYN5.

A number of physiological processes are regulated by hormones, such as senescence [33]. Abscisic acid is essential in hastening leaf senescence through transcriptional regulation [34]. By contrast, auxin, particularly IAA, plays a critical role in the suppression of leaf senescence [35]. In this study, the contents of ABA and IAA were significantly affected by HS, particularly in severe HS treatment (Fig. 7). High concentrations of ABA under stress can serve as inhibitors and negatively affect the photosynthetic antenna absorption and electron transfer on the PSII receptor side [36]. Conversely, high IAA levels drive the maximum leaf gas exchange by regulating leaf stomatal formation and leaf venation, thereby affecting the maximum photosynthetic rate [15]. Further correlation analysis revealed that ABA was positively correlated with MDA and ROS but negatively correlated with SOD, APX, RuBPCase, PEPCase, Pn and Gs, whereas IAA had the opposite correlations. The hormone content of SYN5 was more disordered than that of YN7 under most HS conditions.

**Fig. 9:**
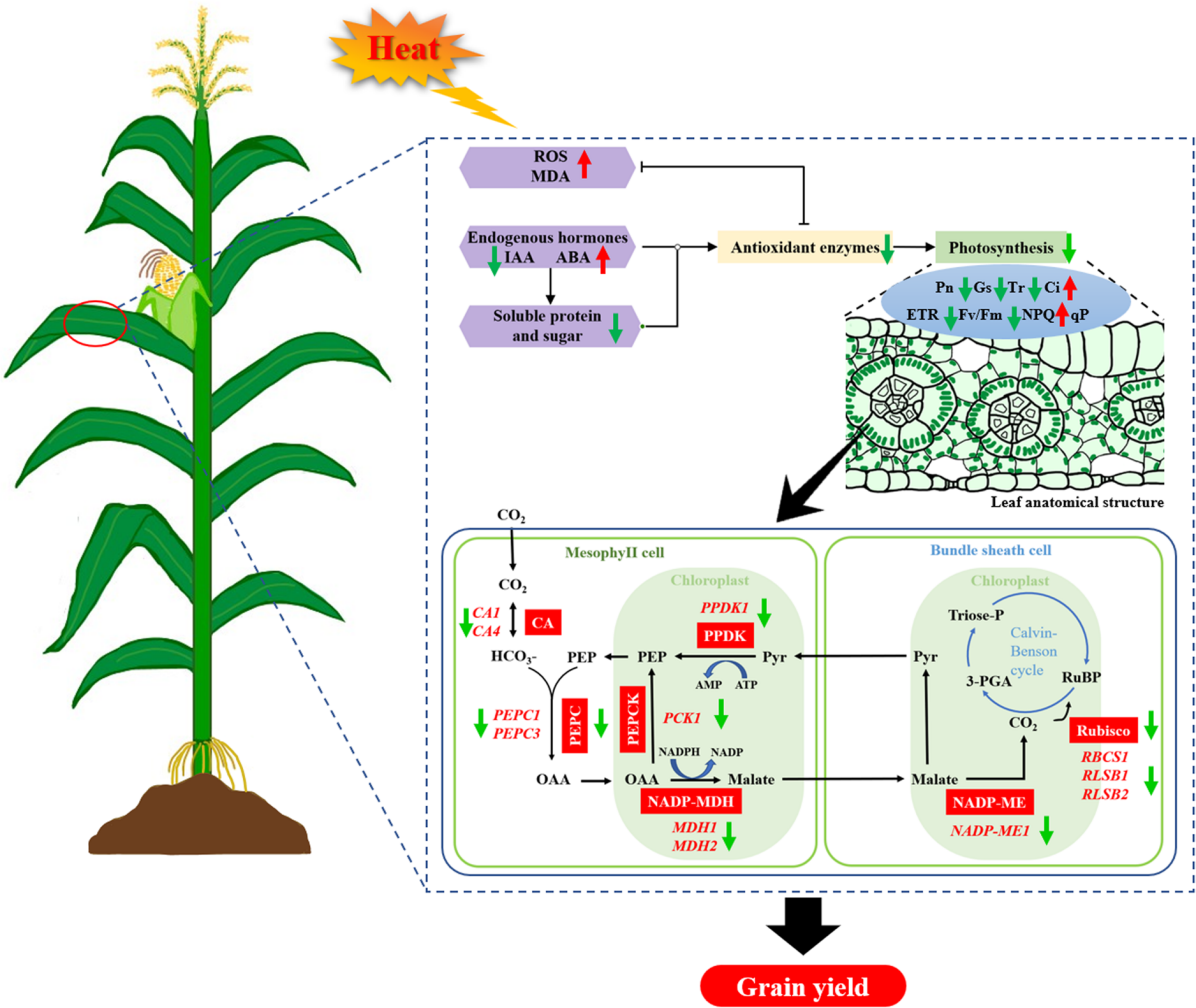
A schematic diagram model for the response of photosynthesis of waxy maize leaves to HS during grain-filling stage. The green arrow indicates a decline, whereas the red arrow indicates an increase

The process of photosynthesis in the leaves creates the energy required for plant growth and development. Light is captured and transformed into chemical energy, which is stored in sugars and transmitted to the other plant organs to satisfy their energy needs [37]. Temperatures above optimal inhibit photosynthesis significantly, resulting in substantial productivity loss [38]. Heat stress significantly inhibited the yield of waxy maize, which was closely related to changes in Pn, soluble protein content, and activities of photosynthetic (RuBPCase and PEPCase) and antioxidant (SOD and POD) enzymes in the present study (Fig. S4). Heat stress accelerates leaf senescence, leading to impaired photosynthetic performance and resulting in the lack of supply of photosynthetic assimilates, which leads to compromised seed filling and reduced yield (Fig. 9). Both waxy maize varieties showed the greatest degree of impact of severe HS (40 °C/32°C) treatment, followed by moderate HS (36 °C/28°C), and the lowest degree of impact of mild HS (32 °C/24°C). Moderate and severe HS increased the accumulation of ROS and ABA in the leaves of waxy maize, resulting in a metabolic imbalance. Meanwhile, the activities of SOD, POD, CAT, and APX were inhibited, further exacerbating the oxidative damage and aging of leaves. The accelerated degradation of chlorophyll and intensified oxidative damage in turn lead to a decrease in leaf photosynthetic performance, ultimately resulting in a decrease in the yield of waxy maize. Although we observed a faster decrease in chlorophyll content in YN7 leaves compared to SYN5 under HS treatments, the overall analysis found that YN7 more resistant to HS. The reason may be that YN7 has more stable hormone levels, higher antioxidant and photosynthetic capacities than SYN5 under HS, which is more conducive to stress resistance production.

## Conclusion

Heat stress affects the expression level of genes and the activity of photosynthetic enzymes, thereby affecting photosynthetic characteristics. The destruction of the antioxidant system and imbalance of hormone content under HS accelerate the senescence of the leaves. Heat stress during grain formation accelerates leaf senescence and impairs photosynthetic characteristics, thereby hindering the accumulation of photo-assimilates and reducing the yield of waxy maize. Amongst the effects of different treatments on waxy maize, 40 °C/32°C (T3, severe HS) had the greatest inhibitory effect, followed by 36 °C/28°C (T2, moderate HS), and 32 °C/24°C (T1, mild HS) had the smallest inhibitory effect. Compared with SYN5, the physiological indexes of YN7 under HS were subjected to less fluctuation, indicating that it had higher heat tolerance. In order to promote a stable yield of waxy maize under future climate warming, this study suggested that YN7 with stronger heat tolerance should be selected as a variety.

### Methods

#### Plant materials and experimental design

In the spring of 2019 and 2020, a pot trial was conducted at the Yangzhou University Experimental Farm (Yangzhou, China) by using Suyunuo5 (SYN5) and Yunuo7 (YN7) as materials. Thirty kilograms of sandy loam soil that had been sieved were placed in each pot (h = 38 cm, d_top_=41 cm, d_botton_=35 cm). Each treatment included 50 pots. The soil properties in this study were the same as that previously reported [7]. In this experiment, seedlings were raised in a seeding tray and then transplanted into a pot. At transplantation, 10 g of compound fertiliser (N/P_2_O_5_/K_2_O = 15%/15%/15%) and 6.6 g of urea (*N* = 46%) were given to each pot (two plants at the seedling stage and one plant remaining at the jointing stage). Before treatments, the plants were grown in the field environment. After manual pollination at the silking stage, the pots were transferred to the intelligent greenhouse (maintained by using an intelligent control system, Fig. S1) for temperature treatments for 1–15 days after pollination (DAP). In this experiment, four temperature treatments were set, including 28 °C (day) /20°C (night; T0, control), 32 °C/24°C (T1, mild HS), 36 °C/28°C (T2, moderate HS) and 40 °C/32°C (T3, severe HS). After HS treatment, the temperature of all greenhouse was adjusted to 28 °C/20°C until maturity.

### Photosynthesis gas exchange parameters

Photosynthetic characteristics of ear leaves were measured using a portable photosynthetic apparatus system (LI-6400, Li-Cor, USA) from 9:00 am to 12:00 pm on sunny days at 5, 10, 15, 20, 25, and 30 DAPs. The parameters measured included Pn, Tr, Gs and Ci. Each measurement took around 2 min and was done in the middle of the ear leaf. All measurements were performed in triplicate.

### Chlorophyll fluorescence

Use a portable chlorophyll fluorescence meter PAM-2000 (Walz, Effeltrich, Germany) to measure chlorophyll fluorescence parameters of ear leaves with 20 min dark treatment at about 7:00 am at 5, 10, 15, 20, 25, and 30 DAPs. These included Fv/Fm, qP, NPQ and ETR. All measurements were performed in triplicate.

### Chlorophyll, soluble sugars, and soluble protein contents in leaves

Fresh leaf samples of ear leaves were taken at 5, 10, 15, 20, 25, and 30 DAPs, and after wiping off the tissue surface dirt and blotting out the water attached to the leaf surface, a portion of the leaves were directly subjected to the determination of chlorophyll content and soluble protein. The soluble protein and chlorophyll content were determined using the Kemas Brilliant Blue G-250 method and spectrophotometer method, respectively [39, 40]. All samples were analyzed three times using three biological replicates.

### Enzymatic activities and endogenous phytohormones content

At 5, 10, 15, 20, 25, and 30 DAPs, ear leaves were taken placed in liquid nitrogen, and then transferred to an ultra-low temperature refrigerator (-75 °C) for storage, which was used for analysis of physiological and biochemical indexes. Using kits produced by Enzyme-linked Biotechnology Co., Ltd (Shanghai, China), the activities of photosynthesis-related enzymes (RuBPCase, ml022780; PEPCase, ml092968) and antioxidant-related enzymes (SOD, ml503401; POD, ml504872; CAT, ml537039; APX, ml700824), and the contents of MDA (ml094965), ROS (ml236087), and hormones (IAA, ml147100; ABA, ml077235) were analyzed. The OD value of RuBPCase, PEPCase, SOD, CAT, POD, MDA, ROS, IAA and ABA was measured with a microplate reader (Rayto, RT-6100, USA) at 450 nm and quantified using the corresponding standard curve. In addition, the OD value of APX was measured at 290 nm. All enzymatic activities and endogenous hormones contents were performed in triplicate.

### RNA preparation and quantitative real-time PCR (qRT-PCR)

Total RNA was extracted from leaf samples of 5 and 15 DAPs by the FastPure Universal Plant Total RNA Isolation Kit (Vazyme, Nanjing, China). Twelve photosynthesis-related genes (*CA1*, *CA4*, *PEPC1*, *PEPC3*, *MDH2*, *MDH4*, *NADPH-ME1*, *PPDK1*, *RBCS1*, *RLSB1*, *RLSB2* and *PCK1*) were selected for qRT-PCR analysis [41]. Designed qRT-PCR primers sequences using Primer Premier 6.0 software and listed them in Table S1. The qRT-PCR was performed on ABI ViiA™ 7 instrument (Applied Biosystems, CA, USA) using SYBR Master Mix (Vazyme, Nanjing, China). The 2^−∆∆Ct^ method was used to calculate the relative expression levels of the genes, with *GAPDH* serving as a reference gene for internal control. Three biological replicates were performed.

### Grain yield

At the maturity, 9 similar ears were selected from each treatment for grain yield determination. The grains were stripped individually from each ear and dried in the sun. Subsequently, the grain yield (g/plant) per ear was measured, with three ears as a group and three groups for each treatment.

### Statistical analysis

Analysis of Variance (ANOVA) was utilized in SPSS v.19.0 (SPSS Inc., Chicago, IL, USA) to examine the study’s data. Use the least significant difference (LSD) with a *P*-value of less than 0.05 to compare the statistical significance levels. The figures were made by GraphPad Prism 8 (San Diego, CA, USA) and Origin Pro 2021 (Origin Inc., Northampton, MA, USA).

### Supplementary Information

**Additional file 1: Figure S1. **The conditions for temperature treatments. A: The photo of intelligent greenhouses; B: Average daytime and nighttime temperatures for each day during the gradient temperature treatment that lasted 15 days. **Figure S2. **Effect of HS during grain-filling stage on Chlorophyll fluorescence parameters of waxy maize leaves. A, B: ETR, apparent electron transfer rate; C, D: qP, photochemical quenching; E, F: NPQ, non-photochemical quenching; G, H: Fv/Fm, PSII primary maximum light energy use efficiency. T0, control; T1, mild HS; T2, moderate HS; T3, severe HS. Error bars denote standard errors from three replicates, and different letters at the same sampling date indicate significant difference at *P* <0.05. **Figure S3. **Effect of HS during grain-filling stage on soluble protein and sugar content of waxy maize leaves. A, B: soluble protein; C, D: soluble sugar. T0, control; T1, mild HS; T2, moderate HS; T3, severe HS. Error bars denote standard errors from three replicates, and different letters at the same sampling date indicate significant difference at*P* <0.05. **Figure S4.** Correlation analysis between waxy maize yield and various physiological indexes of leaves under different degrees of HS during grain filling. Chl, chlorophyll content; Pn, photosynthetic rate; Gs, stomatal conductance; Tr, transpiration rate; Ci, intercellular CO_2_ concentration; ETR, electron transfer rate; qP, photochemical quenching; NPQ, non-photochemical quenching; Fv/Fm, the photosystem II primary maximum light energy use efficiency; RuBPCase, ribulose bisphosphate carboxylase; PEPCase, phosphoenolpyruvate carboxylase; SP, soluble protein; SS, soluble sugar; MDA, malondialdehyde; ROS, reactive oxygen species; SOD, superoxide dismutase; POD, peroxidase; CAT, catalase; APX, ascorbate peroxidase; IAA, auxin; ABA, abscisic acid. **Table S1.** List of primer sequences used in this study.

## Data Availability

All data is available from the corresponding author (dllu@yzu.edu.cn) upon request.
