# Supplementary material for: Leaf photosynthetic characteristics of waxy maize in response to different degrees of heat stress during grain filling
Source: BMC Plant Biol. 2023 Oct 6;23:469. doi: 10.1186/s12870-023-04482-7 (PMC10557211; doi:10.1186/s12870-023-04482-7)
Supplement: Supplementary file 1 — Additional file 1: Figure S1. The conditions for temperature treatments. A: The photo of intelligent greenhouses; B: Average daytime and nighttime temperatures for each day during the gradient temperature treatment that lasted 15 days. Figure S2. Effect of HS during grain-filling stage on Chlorophyll fluorescence parameters of waxy maize leaves. A, B: ETR, apparent electron transfer rate; C, D: qP, photochemical quenching; E, F: NPQ, non-photochemical quenching; G, H: Fv/Fm, PSII primary maximum light energy use efficiency. T0, control; T1, mild HS; T2, moderate HS; T3, severe HS. Error bars denote standard errors from three replicates, and different letters at the same sampling date indicate significant difference at P <0.05. Figure S3. Effect of HS during grain-filling stage on soluble protein and sugar content of waxy maize leaves. A, B: soluble protein; C, D: soluble sugar. T0, control; T1, mild HS; T2, moderate HS; T3, severe HS. Error bars denote standard errors from three replicates, and different letters at the same sampling date indicate significant difference atP <0.05. Figure S4. Correlation analysis between waxy maize yield and various physiological indexes of leaves under different degrees of HS during grain filling. Chl, chlorophyll content; Pn, photosynthetic rate; Gs, stomatal conductance; Tr, transpiration rate; Ci, intercellular CO2 concentration; ETR, electron transfer rate; qP, photochemical quenching; NPQ, non-photochemical quenching; Fv/Fm, the photosystem II primary maximum light energy use efficiency; RuBPCase, ribulose bisphosphate carboxylase; PEPCase, phosphoenolpyruvate carboxylase; SP, soluble protein; SS, soluble sugar; MDA, malondialdehyde; ROS, reactive oxygen species; SOD, superoxide dismutase; POD, peroxidase; CAT, catalase; APX, ascorbate peroxidase; IAA, auxin; ABA, abscisic acid. Table S1. List of primer sequences used in this study. [file 12870_2023_4482_MOESM1_ESM.docx]

***Supplementary information***

**
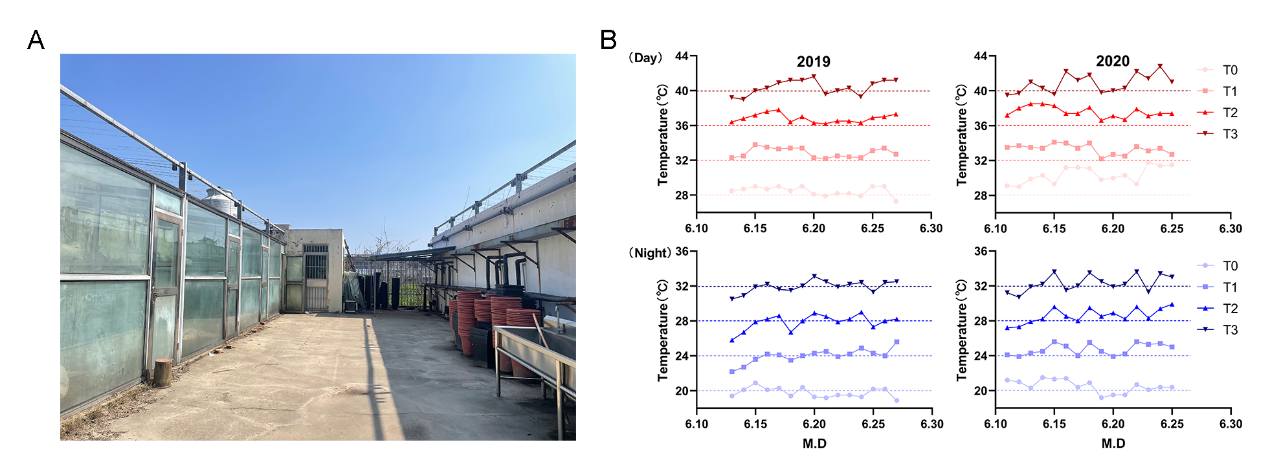
**

**Figure S1.** The conditions for temperature treatments. A: The photo of intelligent greenhouses; B: Average daytime and nighttime temperatures for each day during the gradient temperature treatment that lasted 15 days.


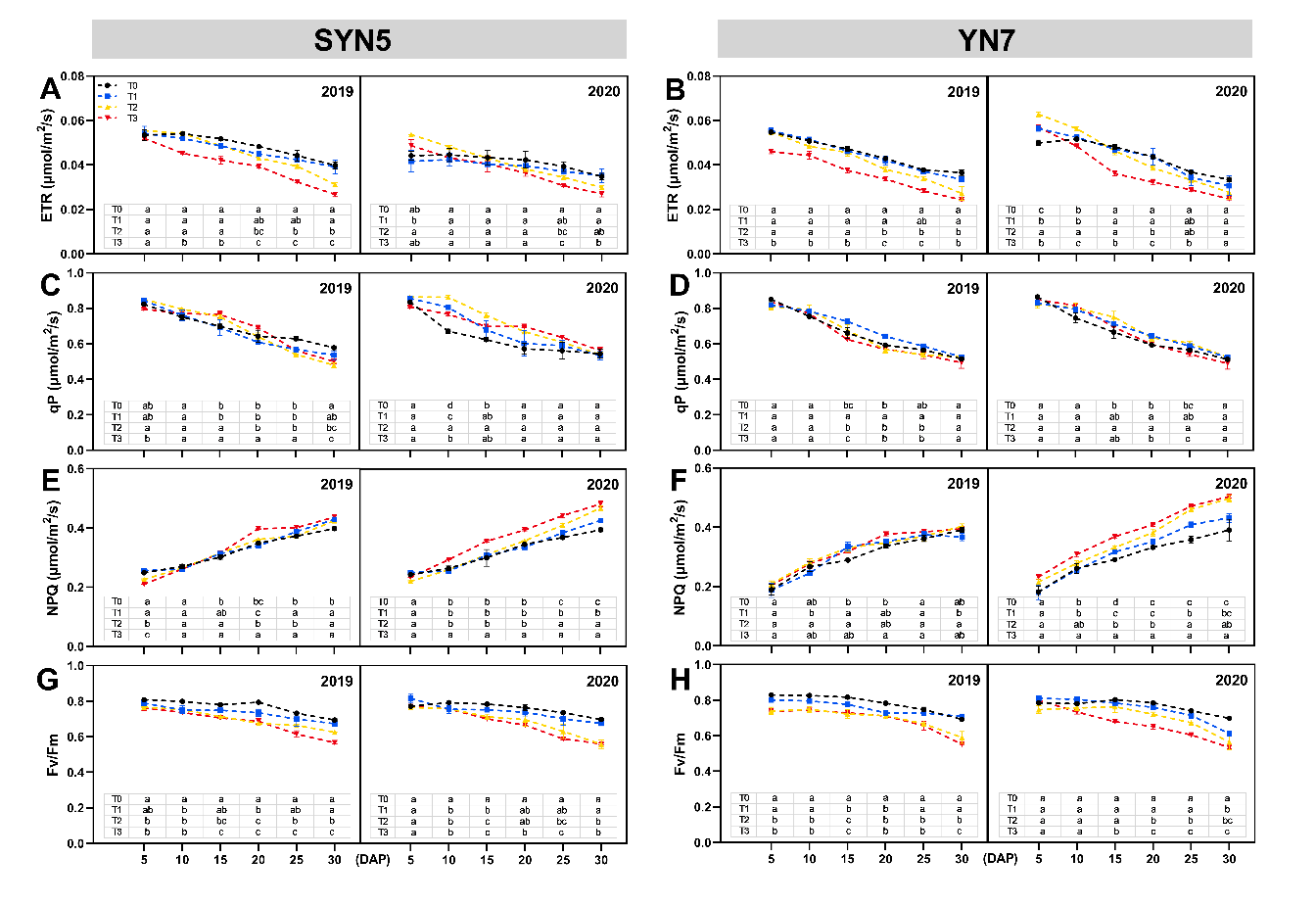


**Figure S2.** Effect of HS during grain-filling stage on Chlorophyll fluorescence parameters of waxy maize leaves. A, B: ETR, apparent electron transfer rate; C, D: qP, photochemical quenching; E, F: NPQ, non-photochemical quenching; G, H: Fv/Fm, PSII primary maximum light energy use efficiency. T0, control; T1, mild HS; T2, moderate HS; T3, severe HS. Error bars denote standard errors from three replicates, and different letters at the same sampling date indicate significant difference at P <0.05.


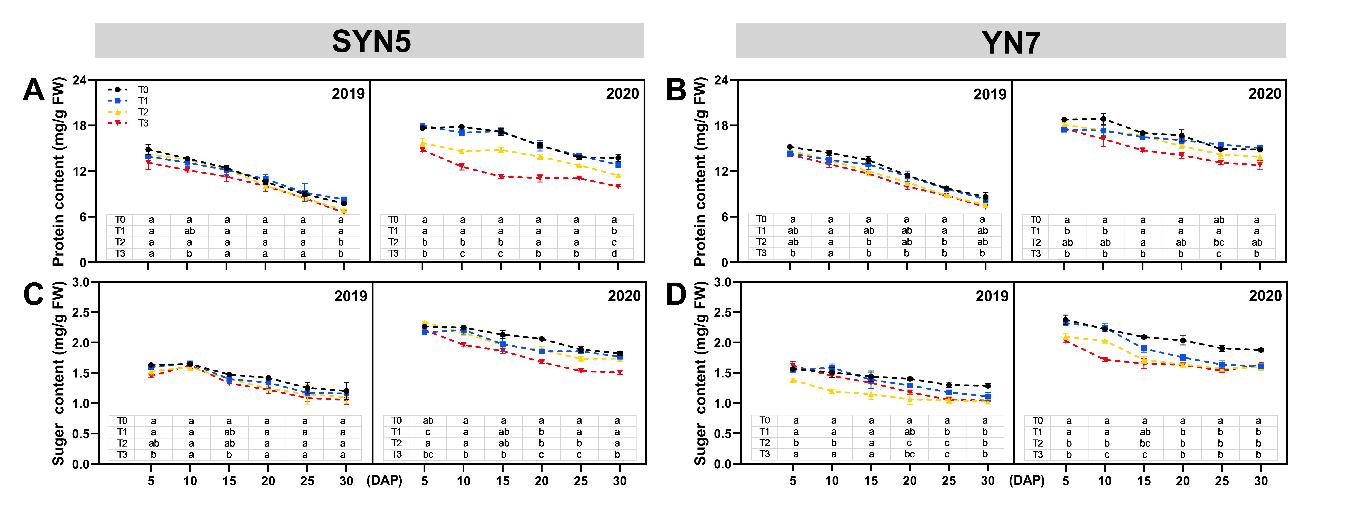


**Figure S3.** Effect of HS during grain-filling stage on soluble protein and sugar content of waxy maize leaves. A, B: soluble protein; C, D: soluble sugar. T0, control; T1, mild HS; T2, moderate HS; T3, severe HS. Error bars denote standard errors from three replicates, and different letters at the same sampling date indicate significant difference at P <0.05


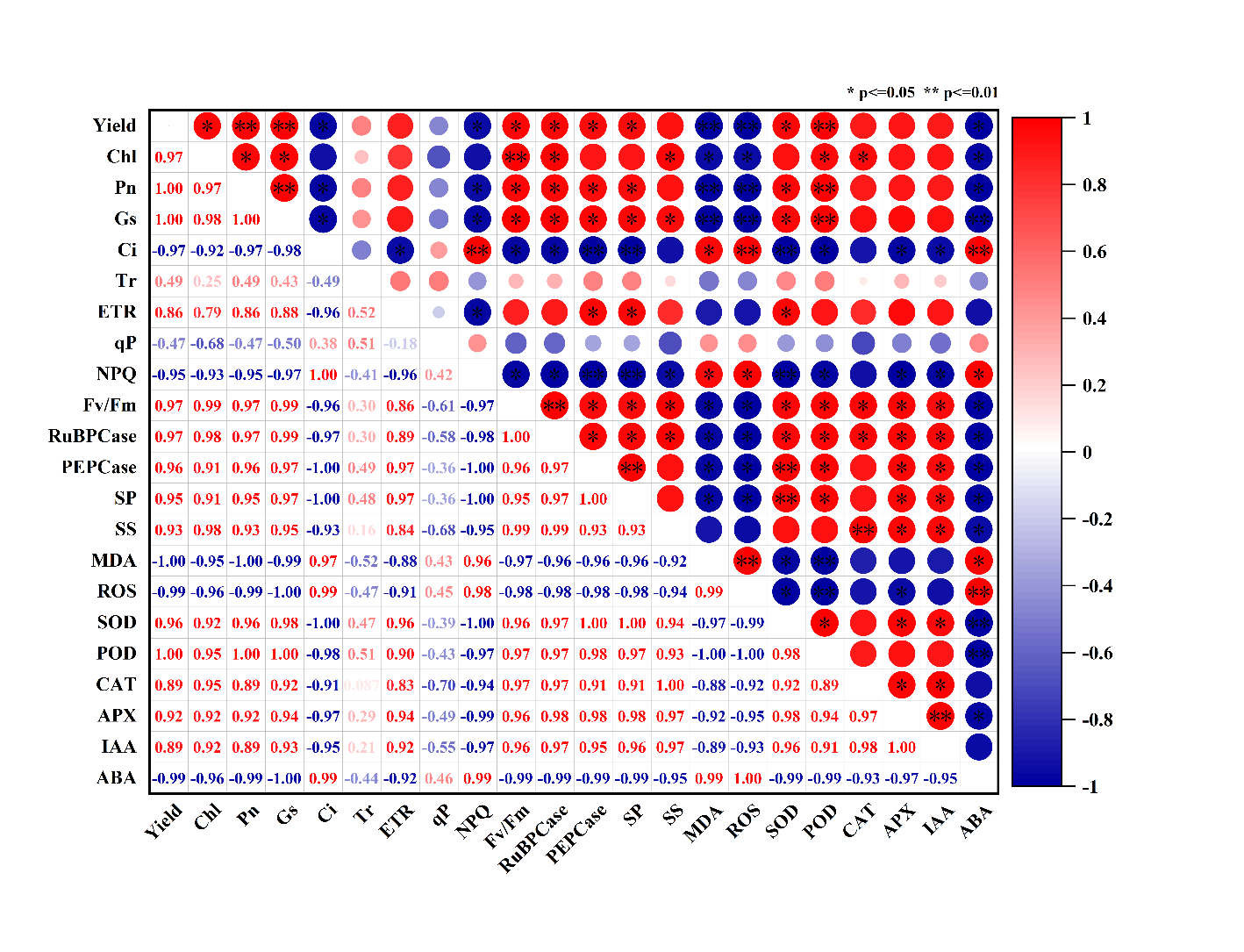


**Figure S4.** Correlation analysis between waxy maize yield and various physiological indexes of leaves under different degrees of HS during grain filling. Chl, chlorophyll content; Pn, photosynthetic rate; Gs, stomatal conductance; Tr, transpiration rate; Ci, intercellular CO_2_ concentration; ETR, electron transfer rate; qP, photochemical quenching; NPQ, non-photochemical quenching; Fv/Fm, the photosystem II primary maximum light energy use efficiency; RuBPCase, ribulose bisphosphate carboxylase; PEPCase, phosphoenolpyruvate carboxylase; SP, soluble protein; SS, soluble sugar; MDA, malondialdehyde; ROS, reactive oxygen species; SOD, superoxide dismutase; POD, peroxidase; CAT, catalase; APX, ascorbate peroxidase; IAA, auxin; ABA, abscisic acid

**Table S1**

**List of primer sequences used in this study**

| **Gene ID** | **Gene Name** | **Description** | **Forward primer (5'-3')** | **Reverse primer (5'-3')** |
| --- | --- | --- | --- | --- |
| Zm00001d044099 | *CA1* | carbonic anhydrase 1 | GCTTGAAGAGCGGGTTCCAG | GGACCATGGAGGCGATGT |
| Zm00001d005920 | *CA4* | carbonic anhydrase 4 | ATTATCTGTCACAATGGCCTCC | AAACCCCAAAACAGCGGTAG |
| Zm00001d046170 | *PEPC1* | phosphoenolpyruvate carboxylase 1 | GCGAATTTGTCCAGGAGTGCT | ACCCACCTTTCTTGAGCTTGC |
| Zm00001d053453 | *PEPC3* | phosphoenolpyruvate carboxylase3 | GGAAGGTCTCCGAGGATGAC | AGGAACTAGCGACGACGATG |
| Zm00001d039089 | *MDH2* | malate dehydrogenase2 | GAAGGGGTTCATGGGGGAC | GACAAGAGCGTTGGGGCAG |
| Zm00001d032695 | *MDH4* | malate dehydrogenase4 | CTGCACATGCTTGACATTCCG | CCTTTCCATTCCCTCCTTCCT |
| Zm00001d037962 | *NADP-ME1* | NADP-dependent malic enzyme2 | AGAGGAACGAGAGGCTTTTCTAC | AGATTCGCTCACCATCAGTAACA |
| Zm00001d038163 | *PPDK1* | pyruvate, orthophosphate dikinase1 | GAGCCATTCCCCTCAGACC | CCATGTTGCCGAACACCAT |
| Zm00001d052595 | *RBCS1* | ribulose bisphosphate carboxylase small subunit1 | CGTTCCAGGGGCTCAAGTC | TGCGCAGCAGGTAGTCCAC |
| Zm00001d044328 | *RLSB1* | rbcl rna s1-binding domain protein1 | GCAGTGAGCATAGCAGACCTTGA | ACCATTGCCAGTTTGCATACAGT |
| Zm00001d038835 | *RLSB2* | rbcl rna s1-binding domain protein2 | AAGTCGGCGAGACGGTGAAA | GGATTCGTCGTTGAAGGGGA |
| Zm00001d028471 | *PCK1* | phosphoenolpyruvate carboxykinase1 | ATGTGCTCAACAACCTCTCGC | AACGTCTTCTCGTCCATCTCG |
| Zm00001d049641 | *GAPDH* | glyceraldehyde-3-phosphate dehydrogenase1 | CCCTTCATCACCACGGACTAC | AACCTTCTTGGCACCACCCT |
